# Supplementary material for: Single rosette-based generation of uniform cortical assembloids recapitulating cellular interactions between neurons and glial cells
Source: Nat Commun. 2025 Nov 25;16:11362. doi: 10.1038/s41467-025-66440-1 (PMC12728168; doi:10.1038/s41467-025-66440-1)
Supplement: Supplementary file 1 — Supplementary Information [file 41467_2025_66440_MOESM1_ESM.pdf]

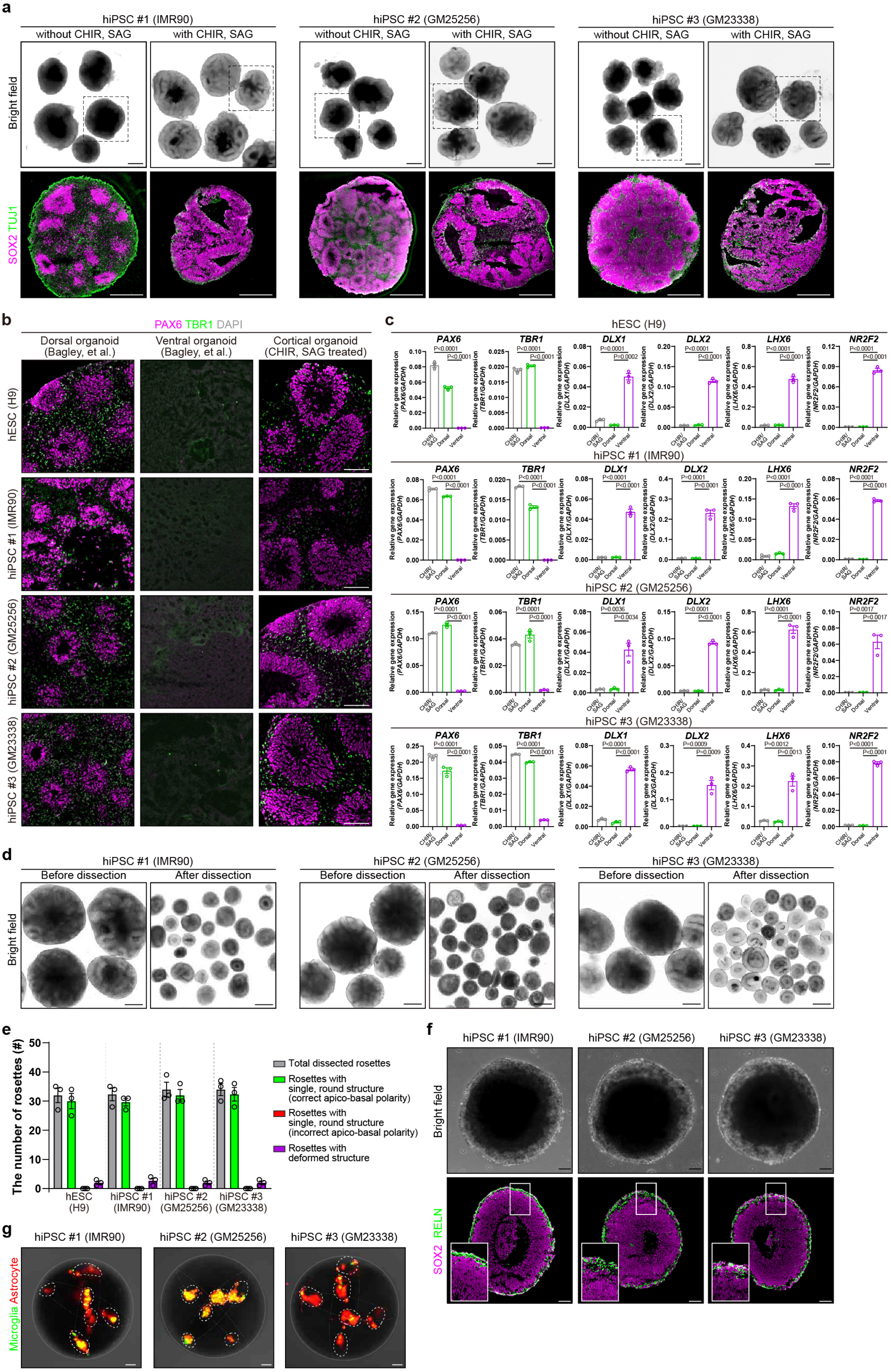
a
hiPSC #1 (IMR90)
hiPSC #2 (GM25256)
hiPSC #3 (GM23338)
without CHIR, SAG
with CHIR, SAG
Bright field
SOX2 TUJ1
b
PAX6 TBR1 DAPI
Dorsal organoid (Bagley, et al.)
Ventral organoid (Bagley, et al.)
Cortical organoid (CHIR, SAG treated)
hESC (H9)
hiPSC #1 (IMR90)
hiPSC #2 (GM25256)
hiPSC #3 (GM23338)
c
hESC (H9)
hiPSC #1 (IMR90)
hiPSC #2 (GM25256)
hiPSC #3 (GM23338)
PAX6
TBR1
DLX1
DLX2
LHX6
NR2F2
Relative gene expression
CHIR/SAG
Dorsal
Ventral
d
hiPSC #1 (IMR90)
hiPSC #2 (GM25256)
hiPSC #3 (GM23338)
Before dissection
After dissection
Bright field
e
The number of rosettes (#)
hESC (H9)
hiPSC #1 (IMR90)
hiPSC #2 (GM25256)
hiPSC #3 (GM23338)
Total dissected rosettes
Rosettes with single, round structure (correct apico-basal polarity)
Rosettes with single, round structure (incorrect apico-basal polarity)
Rosettes with deformed structure
f
hiPSC #1 (IMR90)
hiPSC #2 (GM25256)
hiPSC #3 (GM23338)
Bright field
SOX2 RELN
g
hiPSC #1 (IMR90)
hiPSC #2 (GM25256)
hiPSC #3 (GM23338)
Microglia Astrocyte

Supplementary Fig. 1. Creation of cortical assembloids by a module-based, cellular reconstitution of multiple cell types in human brains

(a) (top) Representative bright field images of early forebrain organoids (day 32), derived from hiPSCs, treated with CHIR99021 and SAG. (bottom) Magnified images of forebrain organoids, demarcated by dotted boxes on top panels, immunostained for NPCs (SOX2) and neurons (TUJ1). Scale bars, 1 mm. (b) Immunostaining analysis of early forebrain organoids (day 32) for PAX6 and TBR1. Scale bars, 100 μm. (c) RT-qPCR analysis for the expressions of *PAX6*, *TBR1*, *DLX1*, *DLX2*, *LHX6*, and *NR2F2*. Data, mean values +/- SEM. (d) Representative bright field images of manually-dissected, single rosettes. Scale bars, 1 mm. (e) Quantitative analysis to assess the proper formation of apico-basal polarity in single rosettes. Data, mean values +/- SEM. (f) Representative images of single rosettes encapsulated with the RELN+ layer (day 35) immunostained for SOX2 and RELN. Scale bars, 100 μm. (g) Representative images of intermediate assembloids (day 50) immediately after being microinjected with glial cells. Astrocytes and microglia were labeled with RFP and GFP, respectively. Scale bars, 100 μm.

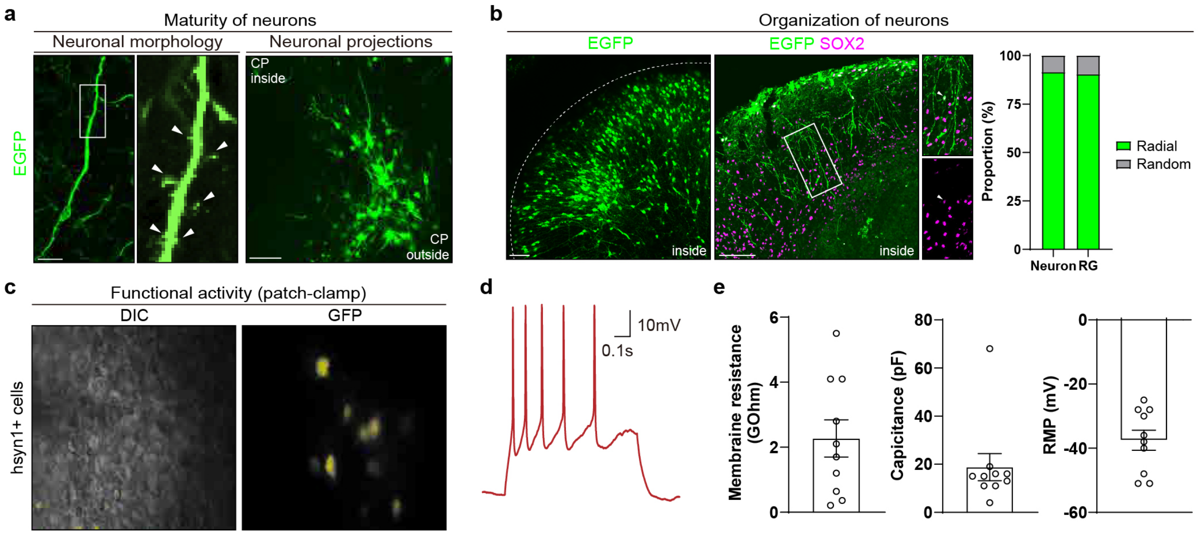

Supplementary Fig. 2. Cortical assembloids represent neuronal maturity at the single-cell level
(a) (left) Representative images of neurons in cortical assembloids (d80), showing neuronal morphology. The white arrowheads indicate dendritic spines. Scale bar, 10 µm. (right) Merged images of a series of z sections of cortical assembloids (d80) with sparsely labeled neurons (EGFP), showing neuronal projections (The sequential scanning video of individual z sections are presented in Supplementary Movie 1). CP; cortical plate. Scale bar, 100 µm. (b) (left) Merged images of a series of z sections of cortical assembloids (d80) with EGFP-labeled neurons and radial glia, showing their radial organization. Scale bar, 100 µm. (middle) Representative images of EGFP-labeled neurons (SOX2-) and radial glia (SOX2+) in cortical assembloids (d80). The white arrowhead indicates radial glia. Scale bar, 100 µm. (right) Quantification of EGFP-labeled neurons (SOX2-) and radial glia (SOX2+) exhibiting radial organization. Cells oriented within ±30° relative to a line drawn perpendicular to the ventricular zone's tangential surface were classified as "radially organized (Radial)", while those outside this range were classified as "randomly organized (Random)". Three sections from cortical assembloids were quantified. (c) Representative images of neurons infected with the AAV-syn1-GFP virus for patch-clamp recording. (d) Representative traces of spontaneous action potential firing in cortical assembloids. (e) Membrane resistance (left), capacitance (middle), and resting membrane potential (right) of a cell from cortical assembloids measured using whole-cell patch-clamp recording. Data, mean values +/- SEM.

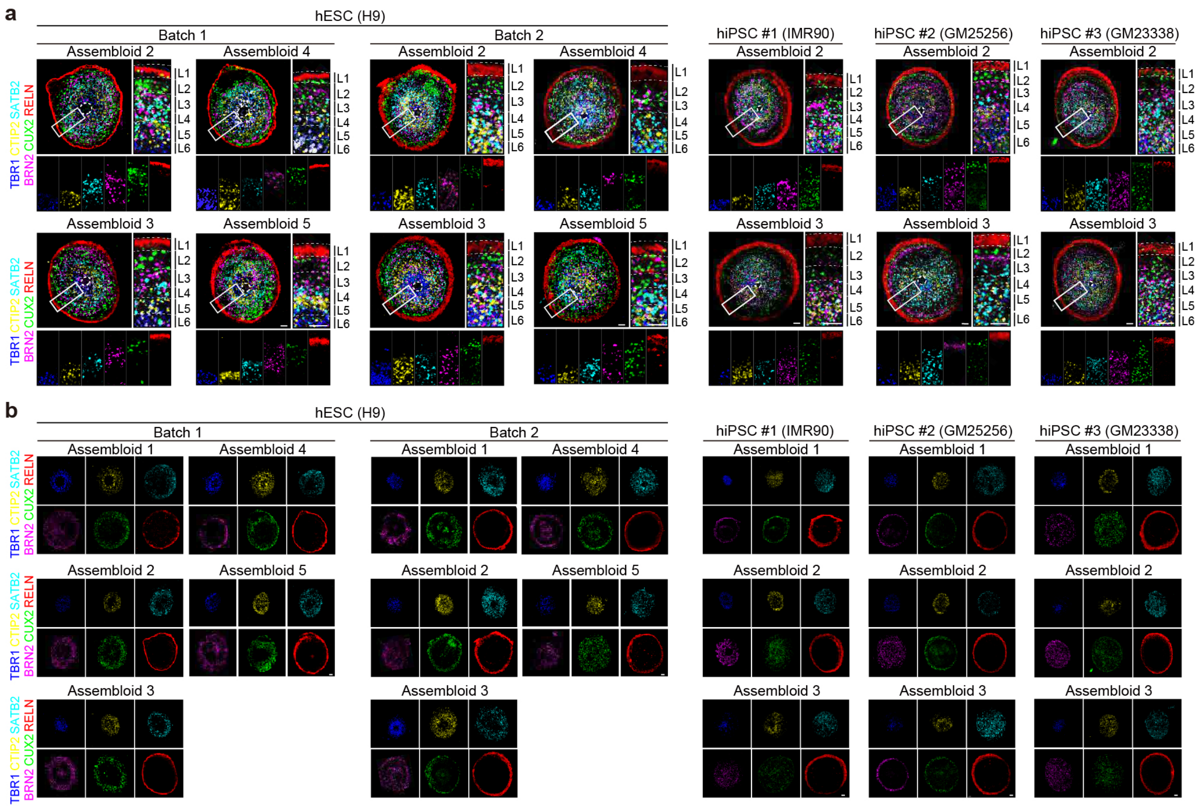

Supplementary Fig. 3. Cortical assembloids represent the six-layered cortical structure

(a) Representative images of 6-layered cortical structures of cortical assembloids (d80). Top left panels show merged images of three serial sections at 8-μm intervals in which each section was immunostained for TBR1/CTIP2, SATB2/RELN, and BRN2/CUX2, respectively. Magnified images (insets in the upper left panels) are shown on the right and bottom panels. Dotted lines demarcate the border of each 6 layer. L1; layer 1, L2; layer 2, L3; layer 3, L4; layer 4, L5; layer 5, L6; layer 6. Scale bars, 100 μm. (b) Images of the TBR1-, CTIP2-, SATB2-, BRN2-, CUX2-, and RELN-positive layers of cortical assembloids (d80) in Fig. 2b and Supplementary Fig. 3a. Scale bars, 100 μm.

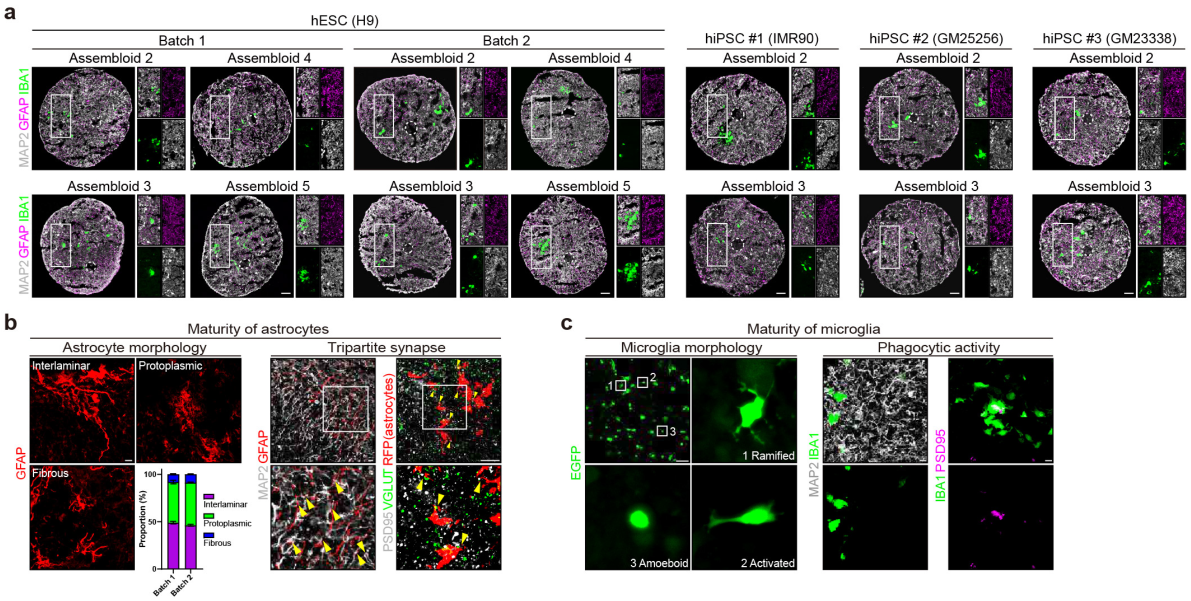

Supplementary Fig. 4. Cortical assembloids contain GFAP-positive astrocytes and IBA1-positive microglia (a) Representative images of cortical assembloids (d80) for neurons (MAP2), astrocytes (GFAP), and microglia (IBA1). Magnified images are shown in the panel on the right. Scale bars, 100 μm. (b) (left) Immunostaining analysis of the morphology of astrocytes in cortical assembloids (d80). Scale bars, 10 μm. GFAP+ astrocytes were categorized into distinct subtypes based on their morphological features. Quantification of these subtypes is shown in the lower right. Cortical assembloids derived from hESC (H9) in two batches were analyzed. Data, mean values +/- SEM. (right) Immunostaining analysis of the function of astrocytes in cortical assembloids (d80). Magnified images (insets in the upper panels) are shown below. The yellow arrowheads indicate tripartite synapses. Scale bars, 10 μm. (c) Immunostaining analysis of the morphology and function of microglia in cortical assembloids (d80). Magnified images for left panels (insets in the upper panel) are shown below. Scale bars, 10 μm.

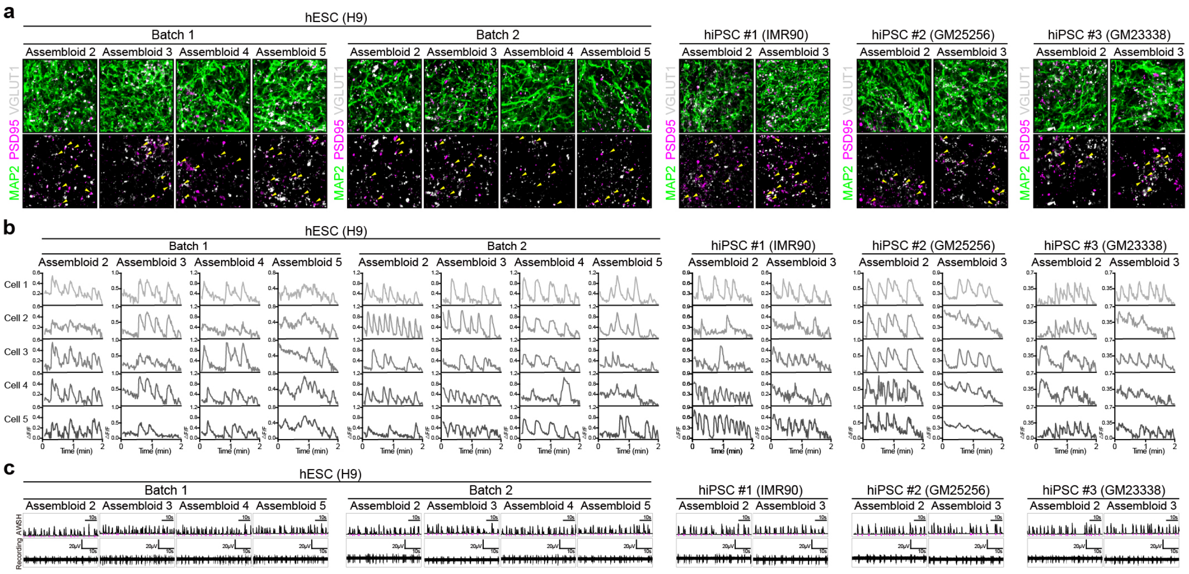

Supplementary Fig. 5. Cortical assembloids display neuronal activity and functional connectivity

(a) Representative images of cortical assembloids (d80) immunostained for neurons (MAP2) and synapses (PSD95 and VGLUT1). The yellow arrowheads indicate synapses co-localized with PSD95 and VGLUT1. Scale bars, 50 μm. (b) Representative images of calcium imaging analyses of selected cells in cortical assembloids (d80). (c) Representative images of the AWSH and recording plot analyzed by MEA in cortical assembloids (d80).

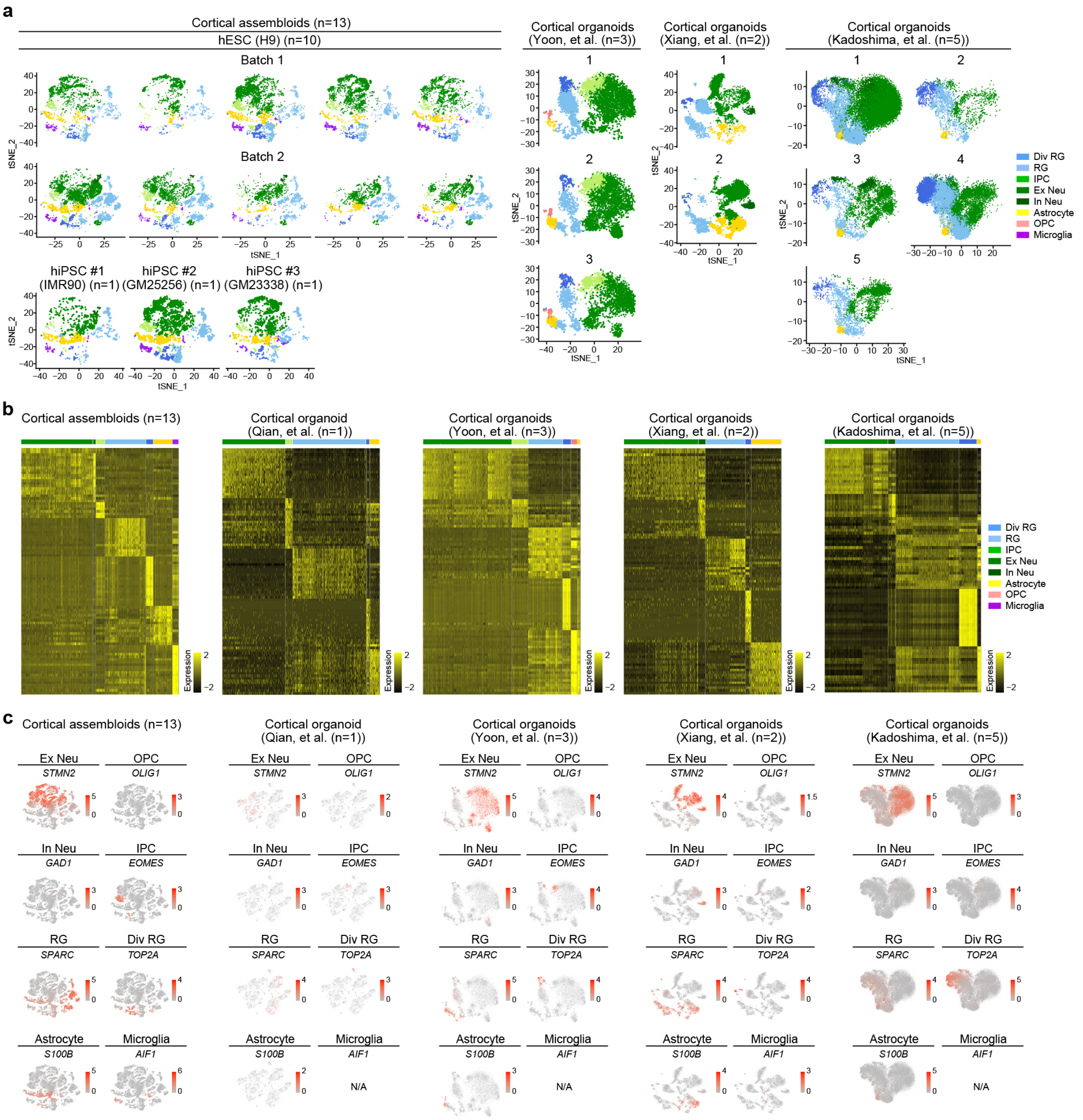

Supplementary Fig. 6. scRNA sequencing analysis for cellular compositions of cortical assembloids in comparison with cortical organoids derived from widely used, four independent protocols

(a) tSNE plots of scRNA-seq data from cortical assembloids and ten independent, widely-utilized forebrain organoids, developed using methods by Yoon, et al., Xiang, et al., and Kadoshima, et al. Cells are colored by cell type and labeled with cell type annotations. RG, radial glia; Div RG, dividing RG; IPC, intermediate progenitor cell; Ex Neu, excitatory neuron; In Neu, inhibitory neuron; OPC, oligodendrocyte progenitor cell. (b) Heatmap representation of the average gene expression of top 20 marker genes expressed in each cell type in current cortical organoids and cortical assembloids. (c) Feature plots showing the expression patterns of key genes used to define major cell type identities.

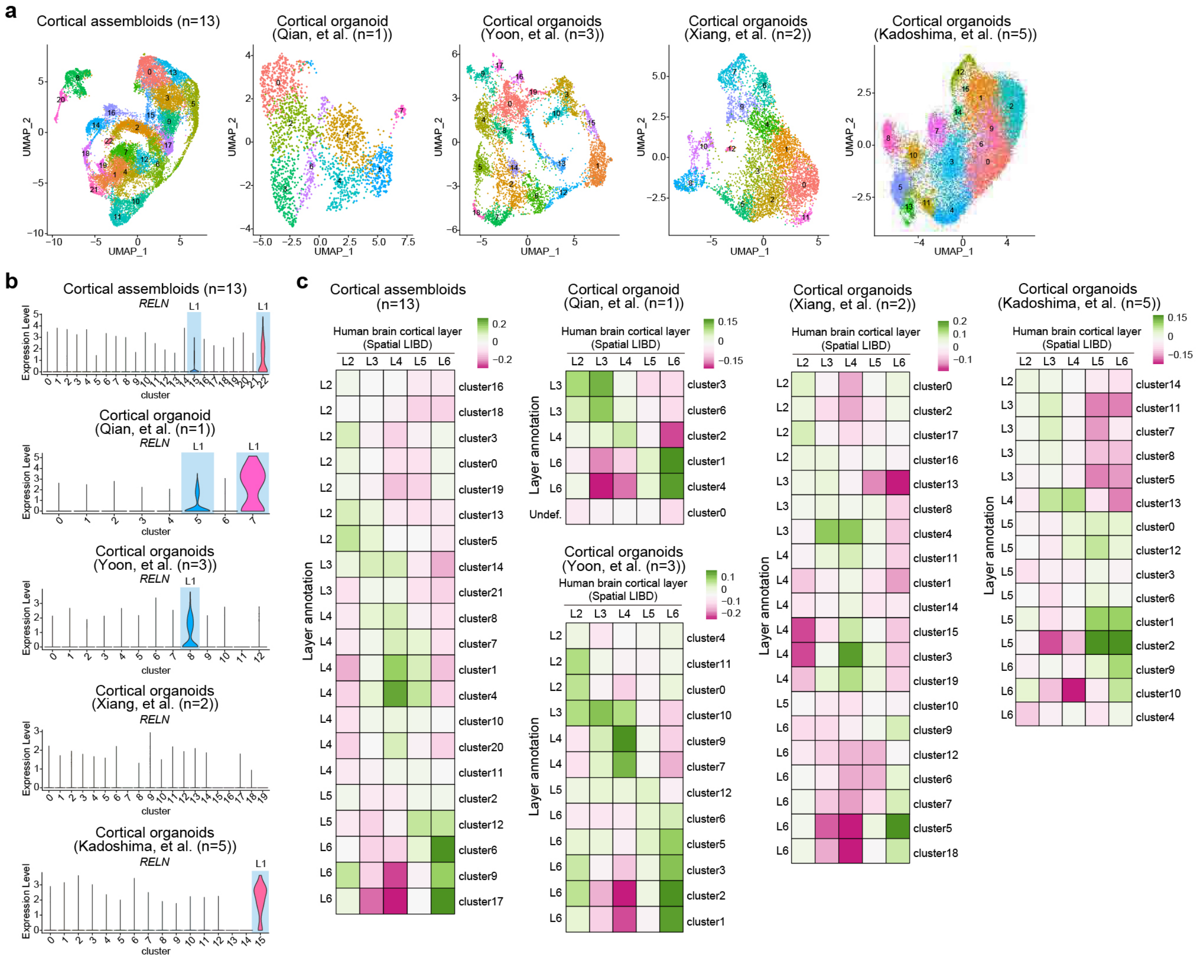

Supplementary Fig. 7. scRNA-seq analysis for cortical layer specification of cortical assembloids in comparison with cortical organoids derived from widely used, four independent protocols

(a) UMAP plots of scRNA-seq data of excitatory neurons from cortical assembloids and eleven independent, widely-utilized cortical organoids, developed using methods by Qian, et al., Yoon, et al., Xiang, et al., and Kadoshima, et al. Cells are colored by cell type and labeled with cell type annotations. (b) Violin plots showing the expression of layer 1 marker gene (*RELN*) in each of excitatory neuron cluster shown in panel 'a'. Clusters highlighted with light blue color were annotated as layer 1. (c) Heatmaps displaying correlation coefficients between excitatory neuron clusters in cortical assembloids/organoids, excluding the clusters annotated as layer 1 in (b), and cortical layers (layer 2-6) in the human brain. Excitatory neuron clusters in assembloids/organoids are re-clustered and further annotated to one of the cortical layers according to their correlation coefficient with the cortical layers in the human brain. Clusters that did not show a positive correlation with any of the six layers in the human brain are designated as 'undefined'. Cells are colored by six cortical layers and labeled with layer annotations.

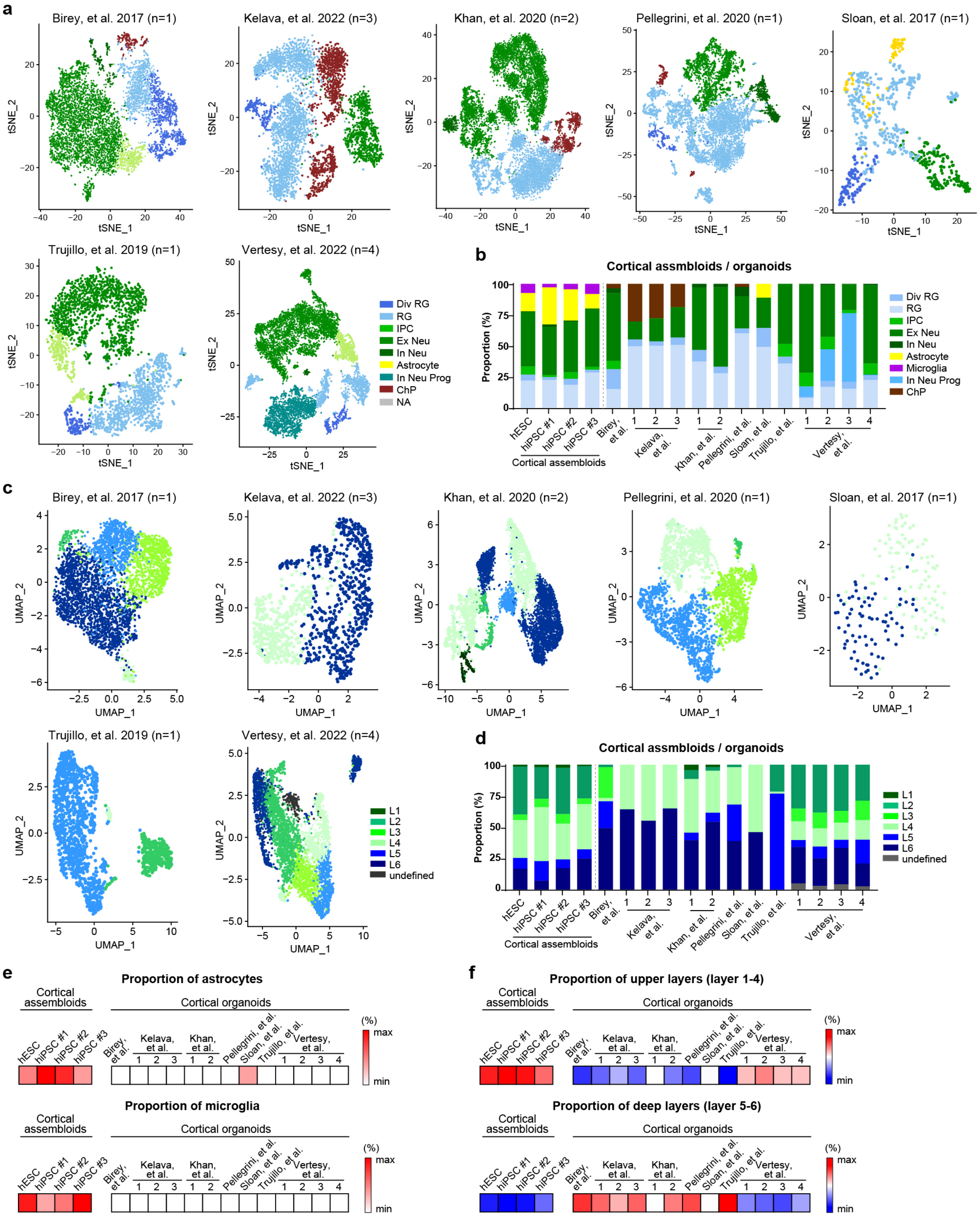

g

Variance

Protocol / Cell type	Cortical assembloids	Birey, et al.	Kelava, et al.	Khan, et al.	Pellegrini, et al.	Sloan, et al.	Trujillo, et al.	Vertesy, et al.
Dividing RG	0.006	N/A	0.022	0.186	N/A	N/A	N/A	0.036
RG	0.001	N/A	0.01	0.143	N/A	N/A	N/A	0.045
IPC	0.007	N/A	N/A	N/A	N/A	N/A	N/A	0.037
Ex Neu	0.002	N/A	0.003	0.050	N/A	N/A	N/A	0.048
In Neu	0.016	N/A	N/A	0.092	N/A	N/A	N/A	0.066
ChP	N/A	N/A	0.028	N/A	N/A	N/A	N/A	N/A
Astrocyte	0.003	N/A	N/A	N/A	N/A	N/A	N/A	N/A
Microglia	0.001	N/A	N/A	N/A	N/A	N/A	N/A	N/A
In Neu Prog	N/A	N/A	N/A	N/A	N/A	N/A	N/A	0.128
Geometric mean	0.003	N/A	0.012	0.105	N/A	N/A	N/A	0.054
Avg	0.006	N/A	0.016	0.118	N/A	N/A	N/A	0.059

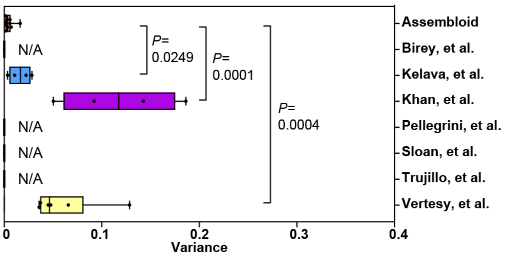

Supplementary Fig. 8. Comparative scRNA sequencing analysis of cortical assembloids using brain organoid atlas datasets

(a) tSNE plots of scRNA-seq data from cortical assembloids and 13 independent organoids from seven scRNA-seq datasets (Birey, et al., Kelava, et al., Khan, et al., Pellegrini, et al., Sloan, et al., Trujillo, et al., Vértesy, et al.). Cells are colored by cell type and labeled with cell type annotations. RG, radial glia; Div RG, dividing RG; IPC, intermediate progenitor cell; Ex Neu, excitatory neuron; In Neu, inhibitory neuron; In Neu Prog, inhibitory neuron progenitor; ChP, choroid plexus cell. (b) Analysis of the proportion of individual cell types in cortical assembloids as well as organoids from seven additional datasets. (c) UMAP plots of scRNA-seq data of excitatory neurons from cortical assembloids and 13 independent organoids from seven scRNA-seq datasets (Birey, et al., Kelava, et al., Khan, et al., Pellegrini, et al., Sloan, et al., Trujillo, et al., Vértesy, et al.). (d) Analysis of the proportion of individual cells according to the six cortical layers in cortical assembloids as well as organoids from seven additional datasets. (e) Comparative analysis of the proportion of astrocytes and microglia in cortical assembloids. The proportions of astrocytes and microglia in cortical assembloids, in comparison to those in organoids, are visualized through heatmaps. (f) Comparative analysis of the proportion of upper-layers and deep-layers in cortical assembloids. The proportions of upper-layers (layer 1-4) and deep-layers (layer 5-6) in cortical assembloids, in comparison to that organoids, are visualized through heatmaps. (g) The variance of each cell type between individual cortical assembloids, as well as among individual organoids from seven additional datasets. Each data point represents the variance of a given cell type within the indicated dataset. Center line, median; whiskers, min to max (show all points).

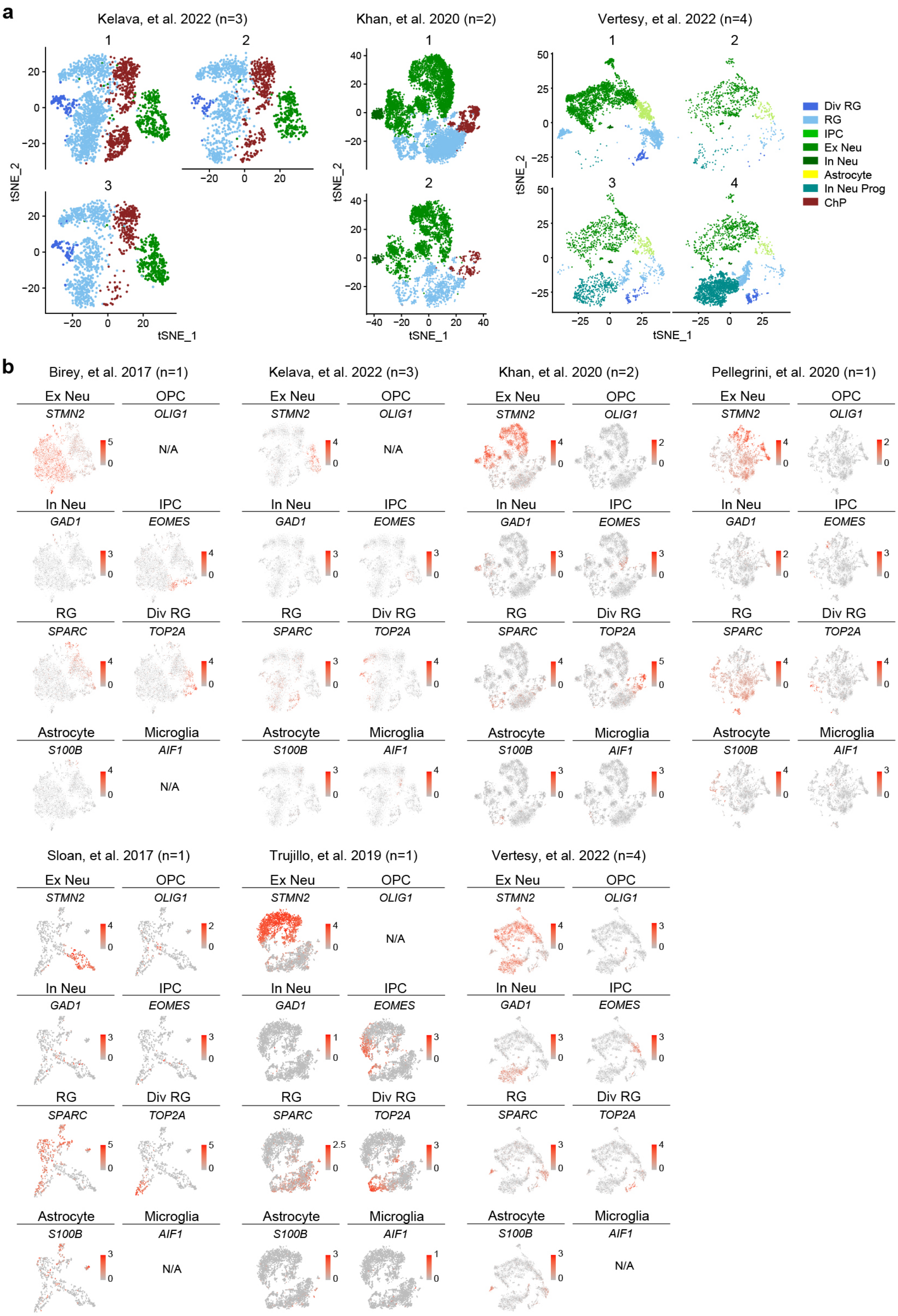

Supplementary Fig. 9. scRNA sequencing analysis for cellular compositions of cortical assembloids using brain organoid atlas datasets

(a) tSNE plots of scRNA-seq data from nine independent organoids from three scRNA-seq datasets (Kelava, et al., Khan, et al., Vértesy, et al.). Cells are colored by cell type and labeled with cell type annotations. RG, radial glia; Div RG, dividing RG; IPC, intermediate progenitor cell; Ex Neu, excitatory neuron; In Neu, inhibitory neuron; In Neu Prog, inhibitory neuron progenitor; ChP, choroid plexus cell. (b) Feature plots showing the expression patterns of key genes used to define major cell type identities.

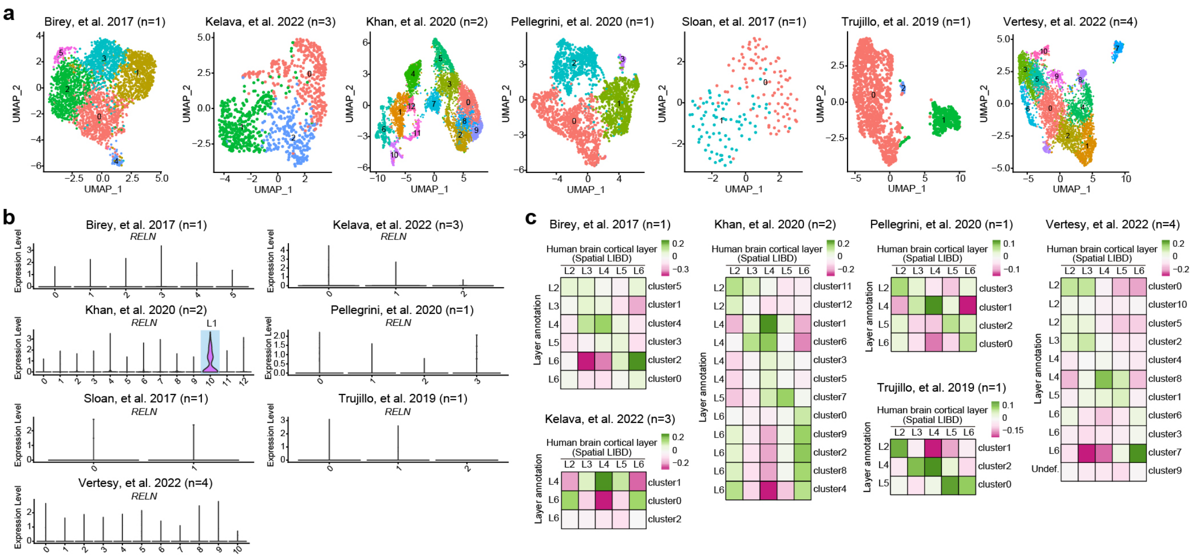

Supplementary Fig. 10. scRNA-seq analysis for cortical layer specification of cortical assembloids using brain organoid atlas datasets

(a) UMAP plots of scRNA-seq data of excitatory neurons from 13 independent organoids from seven scRNA-seq datasets (Birey, et al., Kelava, et al., Khan, et al., Pellegrini, et al., Sloan, et al. Trujillo, et al., Vértesy, et al.). Cells are colored by cell type and labeled with cell type annotations. (b) Violin plots showing the expression of layer 1 marker gene (*RELN*) in each of excitatory neuron cluster shown in panel 'a'. Clusters highlighted with light blue color were annotated as layer 1. (c) Heatmaps displaying correlation coefficients between excitatory neuron clusters in cortical organoids, excluding the clusters annotated as layer 1 in (b), and cortical layers (layer 2-6) in the human brain (heatmaps from Sloan et al. are not shown due to the low number of subclusters—only two—fewer than the minimum three required for correlation). Excitatory neuron clusters in organoids are re-clustered and further annotated to one of the cortical layers according to their correlation coefficient with the cortical layers in the human brain. Clusters that did not show a positive correlation with any of the six layers in the human brain are designated as 'undefined'. Cells are colored by six cortical layers and labeled with layer annotations.

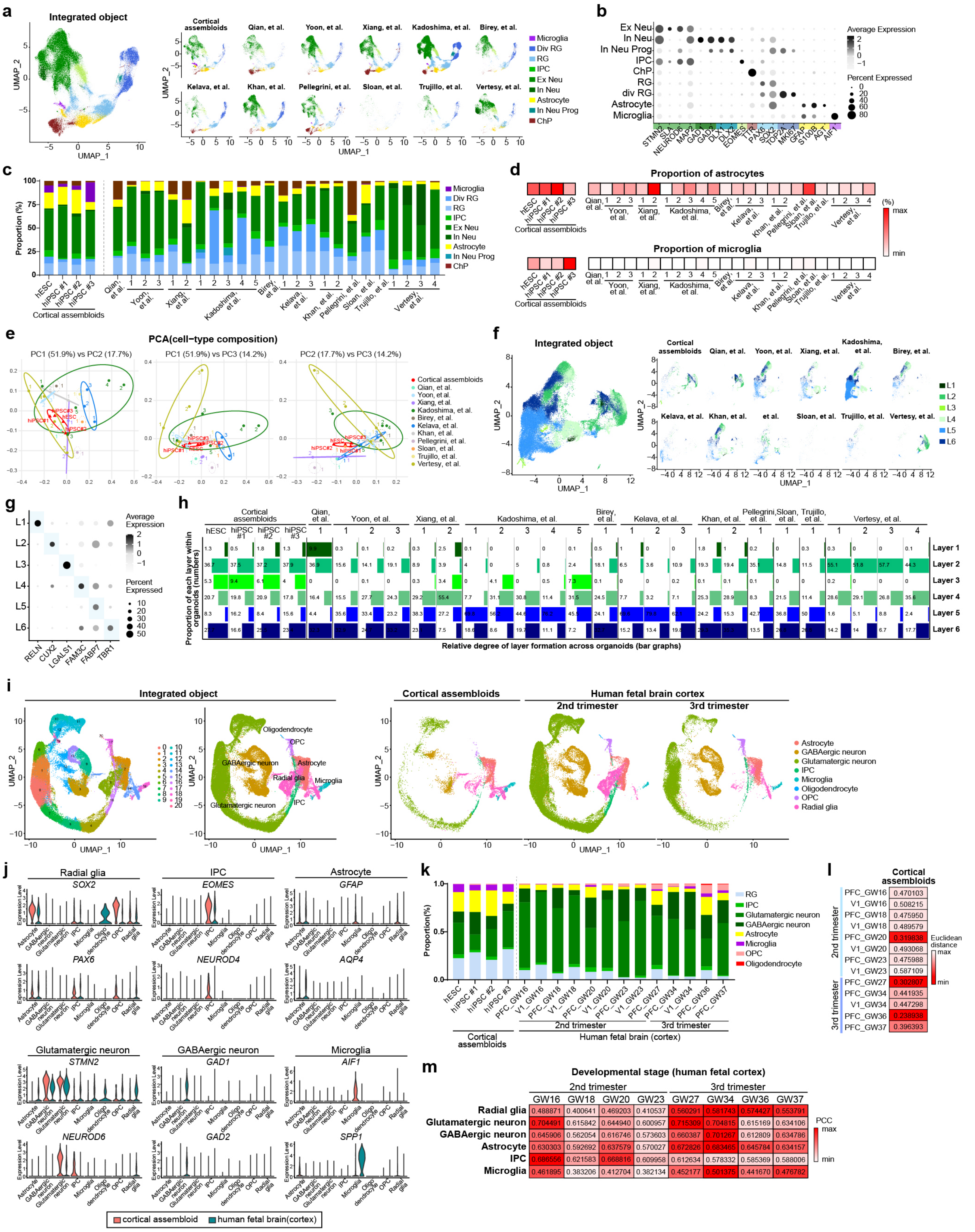

Supplementary Fig. 11. Integrated analysis of cortical assembloids compared with cortical organoids and the developing human brain

(a) UMAP plots of scRNA-seq data from cortical assembloids and cortical organoids from 11 datasets. RG, radial glia; Div RG, dividing RG; IPC, intermediate progenitor cell; Ex Neu, excitatory neuron; In Neu, inhibitory neuron; In Neu Prog, inhibitory neuron progenitor; ChP, choroid plexus cell. (b) Dot plots showing the expression of selected genes across annotated cell types in cortical assembloids and cortical organoids. (c) Analysis of the proportion of individual cell types in cortical assembloids and cortical organoids. (d) Comparative analysis of the proportions of astrocytes and microglia in cortical assembloids and cortical organoids. (e) PCA plots showing the

distribution of cortical assembloids and cortical organoids based on cell-type composition. (f) UMAP plots of scRNA-seq data of excitatory neurons from cortical assembloids and cortical organoids from 11 datasets. L1, layer 1; L2, layer 2; L3, layer 3; L4, layer 4; L5, layer 5; L6, layer 6. (g) Dot plots showing the expression of layer-specific marker genes across the six cortical layers in cortical assembloids and cortical organoids shown in panel 'f'. (h) Bar graphs showing the proportion of excitatory neurons assigned to six cortical layers in cortical assembloids and in cortical organoids. Numerical values within bars indicate the percentage of excitatory neurons assigned to each layer in the corresponding sample. (i) UMAP plots of scRNA-seq data from cortical assembloids and human fetal brain cortex at different developmental stages. IPC, intermediate progenitor cell; OPC, oligodendrocyte precursor cell. (j) Violin plots showing the expression of selected genes across annotated cell types in cortical assembloids and the human fetal brain cortex. (k) Analysis of the proportion of individual cell types in cortical assembloids and human fetal brain cortex. PFC, prefrontal cortex; V1, primary visual cortex; GW, gestational weeks. (l) Heatmap showing Euclidean distances between the cell-type compositions of cortical assembloids and human fetal brain cortex samples. (m) Heatmap of PCCs comparing transcriptomic profiles of matched cell types between cortical assembloids and human fetal brain cortex.
