# Supplementary material for: Single rosette-based generation of uniform cortical assembloids recapitulating cellular interactions between neurons and glial cells
Source: Nat Commun. 2025 Nov 25;16:11362. doi: 10.1038/s41467-025-66440-1 (PMC12728168; doi:10.1038/s41467-025-66440-1)
Supplement: Supplementary file 2 — Description of Addtional Supplementary Files [file 41467_2025_66440_MOESM2_ESM.pdf]

Descriptions of Additional Supplementary Files

Supplementary Movie 1. Sequential scanning video of z section images of cortical assembloids to visualize neuronal projections Sparsely labeled neurons with EGFP were visualized in a sequential scanning video of z section images of cortical assembloids, acquired from confocal microscopy.

Supplementary Movie 2. Live cell imaging analysis for microglia movement in cortical assembloids Movement of EGFP+ microglia in cortical assembloids was visualized using live cell confocal microscopy.

Supplementary Movie 3. Calcium imaging analysis of cortical assembloids at bulk area level Spontaneous calcium surges in neurons throughout the entire area of cortical assembloids, represented by Fluo-4-AM fluorescence, were visualized using live cell confocal microscopy.

Supplementary Movie 4. Calcium imaging analysis of cortical assembloids at single-cell level Spontaneous calcium surges in neurons between connected cortical layers within cortical assembloids, represented by Fluo-4-AM fluorescence, were visualized using live cell confocal microscopy.

Supplementary Data 1. Summary of scRNA-seq analyses of cell clusters identified in cortical assembloids and current brain organoids Per cell type, the overall gene expression, including known marker genes and cell number, analyzed from scRNA-seq data of current brain organoids from eleven datasets and cortical assembloids are listed. Cell types were designated based on the marker genes of each cluster.

Supplementary Data 2. Summary of scRNA-seq analyses of cell clusters identified in cortical assembloids and the developing human brain cortex Per cell type, the overall gene expression, including known marker genes and cell number, analyzed from scRNA-seq data of cortical assembloids and developing human brain cortex (2nd – 3rd trimester) are listed. Cell types were designated based on the marker genes of each cluster.
