# Supplementary material for: Single rosette-based generation of uniform cortical assembloids recapitulating cellular interactions between neurons and glial cells
Source: Nat Commun. 2025 Nov 25;16:11362. doi: 10.1038/s41467-025-66440-1 (PMC12728168; doi:10.1038/s41467-025-66440-1)
Supplement: Supplementary file 9 — Reporting Summary [file 41467_2025_66440_MOESM9_ESM.pdf]

nature portfolio

Corresponding author(s): Kunyoo Shin

Last updated by author(s): Oct 20, 2025

Reporting Summary

Nature Portfolio wishes to improve the reproducibility of the work that we publish. This form provides structure for consistency and transparency in reporting. For further information on Nature Portfolio policies, see our Editorial Policies and the Editorial Policy Checklist.

Statistics

For all statistical analyses, confirm that the following items are present in the figure legend, table legend, main text, or Methods section.

n/a	Confirmed	
☐	☒	The exact sample size (n) for each experimental group/condition, given as a discrete number and unit of measurement
☐	☒	A statement on whether measurements were taken from distinct samples or whether the same sample was measured repeatedly
☐	☒	The statistical test(s) used AND whether they are one- or two-sided<br>*Only common tests should be described solely by name; describe more complex techniques in the Methods section.*
☒	☐	A description of all covariates tested
☒	☐	A description of any assumptions or corrections, such as tests of normality and adjustment for multiple comparisons
☐	☒	A full description of the statistical parameters including central tendency (e.g. means) or other basic estimates (e.g. regression coefficient) AND variation (e.g. standard deviation) or associated estimates of uncertainty (e.g. confidence intervals)
☐	☒	For null hypothesis testing, the test statistic (e.g. F, t, r) with confidence intervals, effect sizes, degrees of freedom and P value noted<br>*Give P values as exact values whenever suitable.*
☒	☐	For Bayesian analysis, information on the choice of priors and Markov chain Monte Carlo settings
☒	☐	For hierarchical and complex designs, identification of the appropriate level for tests and full reporting of outcomes
☐	☒	Estimates of effect sizes (e.g. Cohen's d, Pearson's r), indicating how they were calculated

Our web collection on statistics for biologists contains articles on many of the points above.

Software and code

Policy information about availability of computer code

Data collection	Axion Biosystems Maestro Edge was used to acquire MEA data.<br>Zeiss LSM710 and Nikon AX confocal microscope was used to acquire fluorescence images.<br>Illumina NovaSeq6000 was used to collect scRNA-seq data.

Data analysis	Image J v1.53 was used for calcium imaging analysis.<br>Zeiss Zen and NIS-Elements BR software was used to analyze fluorescence images.<br>GraphPad Prism version 10.0 was used for statistical analyses.<br>AxIS Navigator software was used to analyze MEA data.<br>Clampfit 10.1 software was used to analyze whole-cell patch clamp data.<br><br>scRNA-seq analysis was performed using Ubuntu 22.04.2 LTS, Rstudio (v4.3.1) and Python environment (v3.8.18). Cell Ranger pipeline (v6.1.2) was used to pre-process the 10X sequencing data generated by the 10X Genomics Chromium Controller. The following R packages were used for analysis: Seurat (v4.3.0), scDblFinder (v1.14.0), batchelor (v1.13.3), spatialLIBD (v1.10.1), Scanpy (v1.9.5), dplyr (v1.0.10), patchwork (v1.1.3), ggplot2(v3.3.5), HGNChelper (v0.8.1), bayestestR (v0.13.1), hdf5r (v1.3.8), data.table (v1.14.6), cowplot (v1.1.1), mpmi (v0.43.2.1). All analyses were performed using publicly available packages, tools, and established codes including Seurat (https://satijalab.org/seurat/), Cell Ranger (https://share.google/ouaP1k3vKXVRJ9Ty0), spatialLIBD (https://www.bioconductor.org/packages/release/data/experiment/html/spatialLIBD.html), Scanpy (https://share.google/3t5KpWj0CXlaW3zG4), DESeq2 (https://bioconductor.org/packages/release/bioc/html/DESeq2.html), and mpmi (https://cran.r-project.org/web/packages/mpmi/index.html). Details of the analysis workflow, parameters, and functions used are described in the "Single-cell RNA sequencing" section of the Methods.

For manuscripts utilizing custom algorithms or software that are central to the research but not yet described in published literature, software must be made available to editors and reviewers. We strongly encourage code deposition in a community repository (e.g. GitHub). See the Nature Portfolio guidelines for submitting code & software for further information.

Data

Policy information about availability of data

All manuscripts must include a data availability statement. This statement should provide the following information, where applicable:

- Accession codes, unique identifiers, or web links for publicly available datasets
- A description of any restrictions on data availability
- For clinical datasets or third party data, please ensure that the statement adheres to our policy

TThe scRNA-seq data of cortical assembloids generated in this study have been deposited in the Korea BioData System (K-BDS) database under accession code KAP241776 [https://kbds.re.kr/KAP241776]. Other data generated in this study are provided in the Supplementary Information and Source Data files. The processed scRNA-seq data of the human fetal brain cortex were obtained from the following link: [https://cellxgene.cziscience.com/collections/ad2149fc-19c5-41de-8cfe-44710fbada73]. The scRNA-seq data of previously published cortical organoids from Birey et al., Qian et al., Kadoshima et al., Kelava et al., Khan et al., Pellegrini et al., Qian et al., Sloan et al., Trujillo et al., Vértesy et al., Xiang et al., and Yoon et al. were obtained from the GEO database under the following accession codes, respectively:
GSE93811 [https://www.ncbi.nlm.nih.gov/geo/query/acc.cgi?acc=GSE93811],
GSE132672 [https://www.ncbi.nlm.nih.gov/geo/query/acc.cgi?acc=GSE132672],
GSE187877 [https://www.ncbi.nlm.nih.gov/geo/query/acc.cgi?acc=GSE187877],
GSE145122 [https://www.ncbi.nlm.nih.gov/geo/query/acc.cgi?acc=GSE145122],
GSE150903 [https://www.ncbi.nlm.nih.gov/geo/query/acc.cgi?acc=GSE150903],
GSE137941 [https://www.ncbi.nlm.nih.gov/geo/query/acc.cgi?acc=GSE137941],
GSE99951 [https://www.ncbi.nlm.nih.gov/geo/query/acc.cgi?acc=GSE99951],
GSE130238 [https://www.ncbi.nlm.nih.gov/geo/query/acc.cgi?acc=GSE130238],
GSE205554 [https://www.ncbi.nlm.nih.gov/geo/query/acc.cgi?acc=GSE205554],
GSE98201 [https://www.ncbi.nlm.nih.gov/geo/query/acc.cgi?acc=GSE98201],
GSE107771 [https://www.ncbi.nlm.nih.gov/geo/query/acc.cgi?acc=GSE107771].

Research involving human participants, their data, or biological material

Policy information about studies with human participants or human data. See also policy information about sex, gender (identity/presentation), and sexual orientation and race, ethnicity and racism.

Reporting on sex and gender	*Use the terms sex (biological attribute) and gender (shaped by social and cultural circumstances) carefully in order to avoid confusing both terms. Indicate if findings apply to only one sex or gender; describe whether sex and gender were considered in study design; whether sex and/or gender was determined based on self-reporting or assigned and methods used.*<br>*Provide in the source data disaggregated sex and gender data, where this information has been collected, and if consent has been obtained for sharing of individual-level data; provide overall numbers in this Reporting Summary. Please state if this information has not been collected.*<br>*Report sex- and gender-based analyses where performed, justify reasons for lack of sex- and gender-based analysis.*
Reporting on race, ethnicity, or other socially relevant groupings	*Please specify the socially constructed or socially relevant categorization variable(s) used in your manuscript and explain why they were used. Please note that such variables should not be used as proxies for other socially constructed/relevant variables (for example, race or ethnicity should not be used as a proxy for socioeconomic status).*<br>*Provide clear definitions of the relevant terms used, how they were provided (by the participants/respondents, the researchers, or third parties), and the method(s) used to classify people into the different categories (e.g. self-report, census or administrative data, social media data, etc.)*<br>*Please provide details about how you controlled for confounding variables in your analyses.*
Population characteristics	*Describe the covariate-relevant population characteristics of the human research participants (e.g. age, genotypic information, past and current diagnosis and treatment categories). If you filled out the behavioural & social sciences study design questions and have nothing to add here, write "See above."*

Recruitment	*Describe how participants were recruited. Outline any potential self-selection bias or other biases that may be present and how these are likely to impact results.*
Ethics oversight	*Identify the organization(s) that approved the study protocol.*

Note that full information on the approval of the study protocol must also be provided in the manuscript.

Field-specific reporting

Please select the one below that is the best fit for your research. If you are not sure, read the appropriate sections before making your selection.

☒ Life sciences ☐ Behavioural & social sciences ☐ Ecological, evolutionary & environmental sciences

For a reference copy of the document with all sections, see nature.com/documents/nr-reporting-summary-flat.pdf

Life sciences study design

All studies must disclose on these points even when the disclosure is negative.

Sample size	No statistical methods were used to predetermine sample size. We considered a sample size of at least n=3 to be sufficient for all experiments to ensure the robustness of our conclusions in this study.
Data exclusions	For scRNA-seq, we excluded low quality cells based on criteria as described in the Methods section. No other data were excluded.
Replication	scRNA-seq was performed once. All other experiments were performed at least 3 times independently and all attempts for replication were successful.
Randomization	For all in vitro experiments, samples were randomly assigned to each experimental groups.
Blinding	Investigators were not blinded to allocation during experiments and assessment of results and data. Blinding was not possible as the same investigator performed the in vivo and in vitro experiments and analyzed the data.

Reporting for specific materials, systems and methods

We require information from authors about some types of materials, experimental systems and methods used in many studies. Here, indicate whether each material, system or method listed is relevant to your study. If you are not sure if a list item applies to your research, read the appropriate section before selecting a response.

Materials & experimental systems

n/a	Involved in the study	
☐	☒	Antibodies
☐	☒	Eukaryotic cell lines
☒	☐	Palaeontology and archaeology
☒	☐	Animals and other organisms
☒	☐	Clinical data
☒	☐	Dual use research of concern
☒	☐	Plants

Methods

n/a	Involved in the study	
☒	☐	ChIP-seq
☒	☐	Flow cytometry
☒	☐	MRI-based neuroimaging

Antibodies

Antibodies used

Antibodies used for immunohistochemistry:
TUJ1 (1:300, BioLegend, #801201)
SOX2 (1:300, Millipore, #ab5603)
CTIP2 (1:300, Abcam, #ab18465)
CUX2 (1:300, Abcam, #ab216588)
SATB2 (1:300, Abcam, #ab34735)
TBR1 (1:300, Abcam, #ab31940)
MAP2 (1:300, Abcam, #ab5392)
GFAP (1:300, Dako, #Z0334)
IBAI (1:60, Santacruz, #sc-32725)
RELN (1:200, MBL, #D223-3)
BRN2 (1:300, Santacruz, #sc-393324)
PSD95 (1:300, Invitrogen, #51-6900)
VGLUT1 (1:100, Santacruz, #sc-377425)
ZO-1 (1:200, Santacruz, #sc-33725)
P73 (1:200, Thermo, #PA5-28931)

PAX6 (1:30, DSHB, #AB_528427)

Alexa Fluor 488 goat anti-rabbit IgG (H+L) (Invitrogen, #A11008)
Alexa Fluor 488 goat anti-rat IgG (H+L) (Invitrogen, #A11006)
Alexa Fluor 488 goat anti-mouse IgG (H+L) (Invitrogen, #A11001)
Alexa Fluor 488 goat anti-chicken IgG (H+L) (Invitrogen, #A11039)
Alexa Fluor 594 goat anti-rabbit IgG (H+L) (Invitrogen, #A11012)
Alexa Fluor 594 goat anti-rat IgG (H+L) (Invitrogen, #A11007)
Alexa Fluor 594 goat anti-mouse IgG (H+L) (Invitrogen, #A11005)
Alexa Fluor 594 goat anti-chicken IgG (H+L) (Invitrogen, #A11042)
Alexa Fluor 633 goat anti-rabbit IgG (H+L) (Invitrogen, #A21070)
Alexa Fluor 633 goat anti-rat IgG (H+L) (Invitrogen, #A21094)
Alexa Fluor 633 goat anti-mouse IgG (H+L) (Invitrogen, #A21050)
Alexa Fluor 647 goat anti-chicken IgG (H+L) (Invitrogen, #A21449)

Validation

All primary antibodies and secondary antibodies were validated by the respective manufacturers and further validated in many different published studies.

Eukaryotic cell lines

Policy information about cell lines and Sex and Gender in Research

Cell line source(s)

H9 hESC and IMR90 (healthy, female) hiPSC were obtained from WiCell.
GM25256 (healthy, male) and GM23338 (healthy, male) hiPSCs were obtained from Coriell Institute.

HEK293T cells were obtained from ATCC.

Authentication

All lines were authenticated by manufacturers.

Mycoplasma contamination

The cell lines used in this study were tested for mycoplasma contamination and cell lines used in this study are negative for mycoplasma.

Commonly misidentified lines
(See ICLAC register)

No cell line used in the paper is listed in ICLAC database.

Plants

Seed stocks

Report on the source of all seed stocks or other plant material used. If applicable, state the seed stock centre and catalogue number. If plant specimens were collected from the field, describe the collection location, date and sampling procedures.

Novel plant genotypes

Describe the methods by which all novel plant genotypes were produced. This includes those generated by transgenic approaches, gene editing, chemical/radiation-based mutagenesis and hybridization. For transgenic lines, describe the transformation method, the number of independent lines analyzed and the generation upon which experiments were performed. For gene-edited lines, describe the editor used, the endogenous sequence targeted for editing, the targeting guide RNA sequence (if applicable) and how the editor was applied.

Authentication

Describe any authentication procedures for each seed stock used or novel genotype generated. Describe any experiments used to assess the effect of a mutation and, where applicable, how potential secondary effects (e.g. second site T-DNA insertions, mosiacism, off-target gene editing) were examined.
